# Deregulation of transcription factors controlling intestinal epithelial cell differentiation; a predisposing factor for reduced enteroendocrine cell number in morbidly obese individuals

**DOI:** 10.1038/s41598-017-08487-9

**Published:** 2017-08-15

**Authors:** Bettina K. Wölnerhanssen, Andrew W. Moran, Galina Burdyga, Anne Christin Meyer-Gerspach, Ralph Peterli, Michael Manz, Miriam Thumshirn, Kristian Daly, Christoph Beglinger, Soraya P. Shirazi-Beechey

**Affiliations:** 1 Department of Clinical Research, St. Claraspital Basel, 4058 Basel, Switzerland; 2 Department of Biomedicine, University Hospital Basel, 4056 Basel, Switzerland; 3 Institute of Integrative Biology, University of Liverpool, Liverpool, Merseyside L69 7ZB United Kingdom; 4 Department of Surgery, St. Claraspital Basel, 4058 Basel, Switzerland; 5 Department of Gastroenterology, St. Claraspital Basel, 4058 Basel, Switzerland; 6 Department of Gastroenterology, University Hospital Basel, 4056 Basel, Switzerland

## Abstract

Morbidly obese patients exhibit impaired secretion of gut hormones that may contribute to the development of obesity. After bariatric surgery there is a dramatic increase in gut hormone release. In this study, gastric and duodenal tissues were endoscopically collected from lean, and morbidly obese subjects before and 3 months after laparoscopic sleeve gastrectomy (LSG). Tissue morphology, abundance of chromogranin A, gut hormones, α-defensin, mucin 2, Na^+^/glucose co-transporter 1 (SGLT1) and transcription factors, Hes1, HATH1, NeuroD1, and Ngn3, were determined. In obese patients, the total number of enteroendocrine cells (EEC) and EECs containing gut hormones were significantly reduced in the stomach and duodenum, compared to lean, and returned to normality post-LSG. No changes in villus height/crypt depth were observed. A significant increase in mucin 2 and SGLT1 expression was detected in the obese duodenum. Expression levels of transcription factors required for differentiation of absorptive and secretory cell lineages were altered. We propose that in obesity, there is deregulation in differentiation of intestinal epithelial cell lineages that may influence the levels of released gut hormones. Post-LSG cellular differentiation profile is restored. An understanding of molecular mechanisms controlling epithelial cell differentiation in the obese intestine assists in the development of non-invasive therapeutic strategies.

## Introduction

Obesity and its associated conditions, such as type-2 diabetes and cardiovascular disease, are major health threats worldwide. This condition is partly caused by inability to sense satiation after meals. The intestinal epithelium is a site of satiation signal generation, where specialized enteroendocrine (sensor) cells (EECs) respond to nutrients by releasing hormones that control energy homeostasis, food intake and insulin secretion^1, 2^.

EECs, dispersed among the cells lining the intestinal epithelium, represent approximately 1% of the entire gut epithelial cell population, but together they constitute the largest endocrine organ of the body. Traditionally it has been accepted that the discrete cell types that make up the EEC family have a distinct hormonal profile and localisation along the length of the gut^3^. However, recent investigations have shown that the one hormone, one cell dogma may not always be correct, and that there are more complex patterns of gut hormone co-localisation in EECs^4^. In general, cholecystokinin (*CCK*) is released by I-cells predominantly located in the proximal small intestine, whereas peptide YY (*PYY)* and glucagon-like peptide 1 and 2 (*GLP-1, GLP-2*) are secreted predominantly by L-cells residing mostly in the distal gut^5–7^. However, L-cells have also been identified in the duodenum with higher numbers observed in jejunum and ileum^8^. CCK, GLP-1and PYY regulate gastric emptying and food intake, with GLP-1 also functioning as an incretin hormone, stimulating insulin secretion^9, 10^. *Ghrelin* is released by enteroendocrine X/A cells of the stomach, and by open-type EECs of the duodenum, acting as an orexigenic hormone, stimulating appetite and food intake^11–13^.

Absorptive enterocytes, mucus-producing goblet cells and Paneth cells are the other epithelial cells lining the intestine. All four cell types differentiate from common pluripotent stem cells located near the base of the crypt compartment of the intestine. Cell labelling kinetics has indicated that absorptive enterocytes, goblet and enteroendocrine cells migrate up the crypt-villus axis turning over every 3–4 days, whereas Paneth cells migrate downwards to the crypt base being renewed with a much slower turnover of approximately 21 days^14^.

Absorptive enterocytes are the most abundant cell type in the small intestine (~90%), and they participate mainly in transcellular transport of nutrients^15^. Goblet cells are the most copious secretory lineage of the intestinal epithelia, comprising up to 10% of small intestinal epithelial cells^16^. They produce and secrete mucus to provide epithelial cells a protective shield against noxious luminal contents. Paneth cells produce antimicrobial peptides such as defensins that are secreted into the lumen of the intestine^17^.

The Notch pathway plays a critical role in intestinal epithelial cell fate by regulating the choice of absorptive versus secretory lineages. Notch induces stem cells to express Hes1, a transcription factor proposed to be one of the primary mediators of intestinal Notch signals. Hes1 represses HATH1, a specific secretory lineage transcription factor and thus driving cells to become absorptive enterocytes^16^. In Hes1 deficient mice, HATH1 expression is increased leading to fewer absorptive enterocytes and increased goblet and endocrine cells^18^.

Differentiation of EECs is controlled by the sequential expression of HATH1 and two other basic helix-loop-helix transcription factors, Neurogenin 3 (Ngn3) and NeuroD1. HATH 1 is required for specification and segregation of the intestinal secretory lineage (Paneth, goblet and enteroendocrine cells) from the absorptive enterocyte lineage. Ngn3 expression represents the earliest stage of enteroendocrine differentiation and in its absence enteroendocrine cells fail to develop^19^. Subsequent expression of NeuroD1 appears to represent a later stage of differentiation for maturing EECs. Enteroendocrine cell fate is inhibited by the Notch signalling pathway, which appears to inhibit both HATH1 and Ngn3^19^. Recent studies demonstrating increased numbers of EECs, reduced number of goblet cells, and absence of Paneth cells in Gfi-1 transcription factor-null mice propose additional secretory precursors segregating from HATH-1 dependent cells^20^.

Morbidly obese patients exhibit impaired secretion of gut hormones CCK, GLP-1 and PYY that may contribute to the development of obesity. Certain bariatric procedures such as Roux-en-Y gastric bypass (RYGB) and laparoscopic sleeve gastrectomy (LSG) cause weight loss and induce changes in both gut hormone and insulin secretion, together with improvements in glucose metabolism^10, 21, 22^.

LSG is becoming a widely used method in the treatment of morbid obesity. In 2011 almost 30% of all bariatric interventions performed worldwide were LSGs^23^. It has proved to be effective in terms of sustainable weight loss and amelioration of co- morbidities such as arterial hypertension and glucose intolerance, amongst others^24, 25^. This is probably achieved by a combination of reduction in stomach volume capacity, acceleration of food passage and hormonal changes^21, 25–27^. In obese individuals, gut hormone secretion is impaired with lower postprandial levels of circulating CCK, GLP-1 and PYY^28–30^. The concentrations of fasting ghrelin in the majority of obese are lower than normal-weight individuals, and they do not show a calorie-dependent suppression in postprandial circulating ghrelin as observed in normal weight volunteers^31^. Meal stimulation before and after bariatric surgery reveals dramatic increases in circulatory levels of gut hormones such as GLP-1, PYY and CCK as early as one week after intervention, well before weight loss occurs^10^. The effect of increased secretion of GLP-1 and PYY after bariatric surgery is not well understood, but the mechanism underlying this over-secretion in RYGB has been proposed to be due to accelerated travel of nutrients to the distal gut^32^; this proposed mechanism however does not entirely explain increased gut hormone secretion in LSG.

We hypothesised that there is a decrease in the number of EECs in obese subjects and that this decrease contributes to the observed decline in postprandial gut hormone levels. Furthermore, we aimed to understand the causal processes of increased gut hormone release after LSG.

In this study, we first confirmed that there is a significant increase in postprandial GLP-1, PYY and CCK release in a cohort of morbidly obese subjects 3 months post LSG compared to that in the same individuals, pre LSG. A concurrent decrease in circulating ghrelin levels was also seen post LSG. Subsequently, we demonstrated in a separate, but comparable, cohort of morbidly obese subjects, from whom we could secure consent for obtaining stomach and duodenal biopsies, that there is a significant decrease in the total number of EECs in the stomach and the duodenum, compared to lean subjects. EEC numbers were restored post-operatively. There were no changes in villus height and crypt depth between the two groups. Furthermore, in obese individuals, there was a significant increase in the expression levels of the intestinal glucose transporter, SGLT1 (a marker of absorptive enterocytes), and the major component of intestinal mucus, mucin 2 (an indicator of goblet cells), indicating that there is deregulation of processes controlling cellular differentiation. Accordingly, we demonstrated that protein abundance of transcription factors required for differentiation of intestinal absorptive and secretory cell lineages was also altered; these were returned to normality after LSG.

Understanding mechanisms underlying deregulation of intestinal epithelial cell differentiation in obesity may facilitate identifying potential targets for preventing or treating obesity and associated conditions.

## Results

### Circulating gut hormone levels in a cohort of morbidly obese subjects (Group 1) before and after LSG

Analysis of pre- and post-operative data from group 1, consisting of 14 morbidly obese subjects, demonstrated that there was a marked reduction in BMI (p < 0.001), HOMA index (*Homeostasis model assessment*: [fasting insulin × fasting glucose]/22.5; (p = 0.02), fasting insulin (p = 0.04) and fasting glucose (p = 0.008), at 3 months post-LSG (Table 1 and Figure S1). A significant increase in postprandial GLP-1, PYY and CCK-responses were shown with 2.9- (p < 0.001), 5.7- (p < 0.001) and 1.7-fold (p < 0.01) increases in maximum plasma concentrations of CCK, GLP-1 and PYY, respectively, in 3 month post-operative patients (Table 1 and Figure S1). Pre-operatively, area under the curve values for each hormone were significantly lower (Table 1 and Figure S1). A concurrent 1.7-fold (p < 0.001) decrease in circulating ghrelin levels was also seen 3 months post-operative (Table 1 and Figure S1). Our investigations indicated that age did not affect the hormone responses.

**Table 1:** Baseline demographics and gastrointestinal hormone levels in morbidly obese patients (Group 1) pre- and 3 months post-LSG after liquid test meal (408 kcal) stimulation.

Parameter	Preoperative (n = 14)	3 months (n = 14)	P value
**Gender: male/female**	4/10	—	—
**Age (years)**	37 ± 3	—	—
**BMI (kg/m** ^**2**^ **)**	45.5 ± 1.7	39.3 ± 1.4	p ≤ 0.001
**Insulin**			
Fasting insulin (µU/ml)	36.3 ± 6.5	25.0 ± 3.8	p = 0.036
AUC 0–180 min.	15,371.5 ± 3,799.0	10,954.3 ± 1,534.4	p = 0.297
Cmax (µU/ml)	152.3 ± 22.7	182.1 ± 24.9	p = 0.361
**Glucose**			
Fasting glucose (mmol/l)	6.3 ± 0.5	5.3 ± 0.2	p = 0.008
AUC 0–180 min.	1,070.3 ± 64.0	966.7 ± 36.0	p = 0.040
Cmax (mmol/l)	7.8 ± 0.8	7.4 ± 0.5	p = 0.493
HOMA Index	11.2 ± 2.8	6.3 ± 1.3	p = 0.019
**Ghrelin**			
Fasting ghrelin (pg/ml)	504.9 ± 59.6	272.4 ± 13.9	p = 0.001
AUC 0–180 min.	89,822.5 ± 9,034.7	50,690.5 ± 2,961.4	p = 0.001
Cmax (pg/ml)	535.6 ± 56.2	308.2 ± 17.5	p = 0.001
**CCK**			
Fasting CCK (pMol/ml)	1.0 ± 0.1	0.8 ± 0.0	p = 0.231
AUC 0–180 min.	573.3 ± 73.8	777.1 ± 128.3	p = 0.015
Cmax (pMol/ml)	4.6 ± 0.6	13.6 ± 1.2	p < 0.001
**GLP-1**			
Fasting GLP-1 (pg/ml)	1.5 ± 0.4	1.1 ± 0.1	p = 0.243
AUC 0–180 min.	354.8 ± 62.4	959.4 ± 136.0	p = 0.004
Cmax (pg/ml)	2.6 ± 0.4	13.4 ± 1.9	p < 0.001
**PYY**			
Fasting PYY (pg/ml)	113.6 ± 6.9	95.6 ± 8.6	p = 0.059
AUC 0–180 min.	23,141.9 ± 1,212.2	43,329.4 ± 7,206.8	p = 0.022
Cmax (pg/ml)	150.5 ± 7.7	326.8 ± 62.6	p = 0.019

Data represent means ± SEM; AUC = area under the concentration time profile; Cmax = maximum plasma concentrations. p-value: pre versus post-surgery, using a Student’s paired t-test.

### Determination of tissue expression of gut hormones in the stomach and duodenum of morbidly obese subjects (Group 2)

Twenty seven non-diabetic morbidly obese subjects, who consented to provide stomach and duodenal biopsies pre- and post- LSG, were used in this study. There were 6 drop-outs (5 patients did not want to come back after surgery, one patient had an incidental finding of antrum carcinoma and had to be excluded). Of the morbidly obese patients, 21.1% (7/33) were positive for Helicobacter pylori preoperatively. After surgery there was a marked reduction in BMI from 47.2 ± 1.7 kg/m^2^ (range: 35.6–75.7) to 39.3 ± 1.4 kg/m^2^ (range: 27.1–53.2). The control group consisted of 24 healthy lean subjects (Table 2).

**Table 2:** Baseline demographics in (Group 2) morbidly obese patients pre- and 3 months post-LSG and lean controls.

Parameter	Lean controls (n = 24)	Obese preoperative (n = 33)	Obese 3 months after LSG (n = 27)	P value
Male/female	12/12	14/19	12/15	n.s.
Age (years)	32.0 ± 2.1	41.2 ± 2.1	41.3 ± 2.2	p = 0.01^b^
				p = 0.01^c^
BMI (kg/m^2^)	22.8 ± 0.6	47.2 ± 1.7	39.3 ± 1.4	p ≤ 0.001^a^
				p ≤ 0.001^b^
				p ≤ 0.001^c^

Data represent means ± SEM; p-value: ^a^pre versus post-surgery, ^b^non-operated obese vs. lean, ^c^obese post-surgery vs. lean, using a Student’s paired *t*-test.

#### Assessment of chromogranin A- and ghrelin-containing EECs in the stomach

Using immunohistochemistry, we showed that there was a 2.2- (p < 0.001) and 2.7-fold (p < 0.001) decline in the total number of chromogranin A (ChA)-containing EECs in the lesser and greater curvature of the stomach, respectively, in obese compared to lean individuals. In the same obese individuals post-operatively there was a 1.6-fold (p < 0.05) increase in the number of these cells in the lesser curvature compared to pre-LSG (Figure S2 and Table S1). Tissues from the greater curvature were no longer available after surgery to be compared. Using an average cell count of 500, we observed a 2.5- (p < 0.001) and 2.8-fold (p < 0.001) decrease in EECs containing ghrelin in the lesser and greater curvature respectively, in the obese stomach compared to lean. The number of ghrelin-containing cells was increased 1.5-fold (p < 0.05) in lower curvature of post-operative vs. pre-operative obese subjects, however this was still 1.5-fold (p < 0.05) lower than lean controls. We showed that the greater and lesser curvature of the stomach do not contain EECs possessing CCK, GLP-1, GLP-2 or PYY. The total number of cells counted that contain ChA and ghrelin is given in Table S1.

#### Assessment of the number of chromogranin A and gut hormone containing EECs in the duodenum

**Figure 1:**
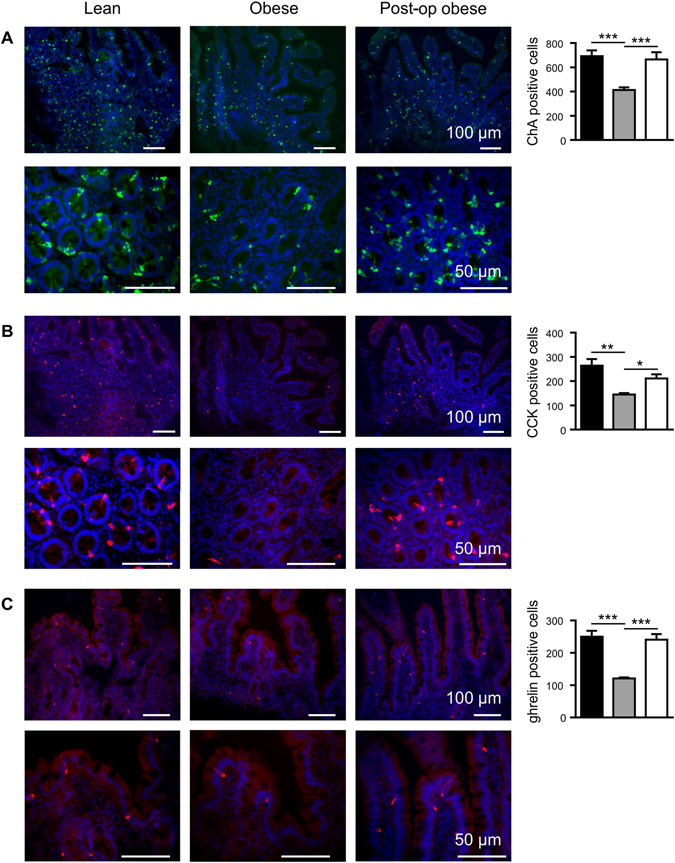
Expression of chromogranin A, CCK and ghrelin proteins in human duodenal biopsies. Profile of the enteroendocrine cell marker, chromogranin A (**A**, ChA) and the gut hormones CCK (**B**) and ghrelin (**C**), was determined in the duodenal biopsies of lean (■), obese () and post-operative obese, (□) by immunohistochemistry. Bar charts (on the right) show number of cells counted expressing ChA or gut hormones. Statistical significance was determined by a One-way ANOVA with differences between means identified using a Holm-Sidak multiple comparison post-test where, *p < 0.05, **p < 0.01 and ***p < 0.001. Scale bars are either 100 or 50 µm. Nuclei are stained blue with 4′,6-diamidino-2-phenylindole (Dapi).

**Figure 2:**
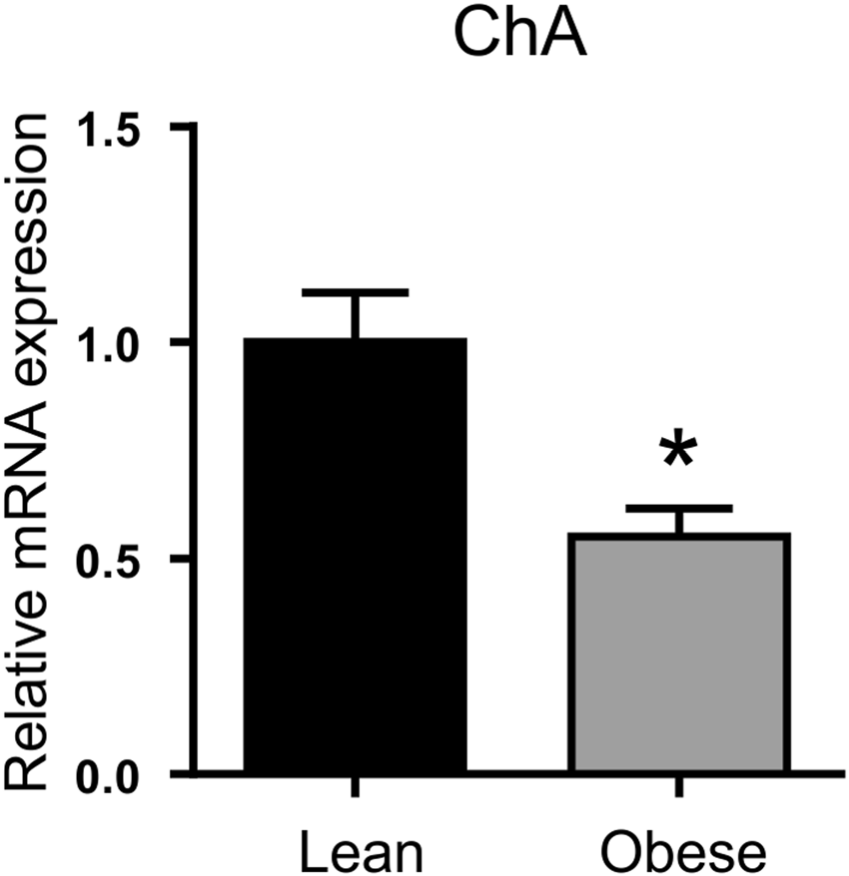
Expression profile of chromogranin A mRNA in duodenal biopsies. Relative mRNA abundance in lean (■) and obese () duodenum for ChA (enteroendocrine cell marker) determined by qPCR. Values are means ± SEM, normalised to RNA polymerase IIA (POLR2A) m RNA expression, n = 4. Statistical significance was determined by a Student’s *t*-test where *p < 0.05.

**Figure 3:**
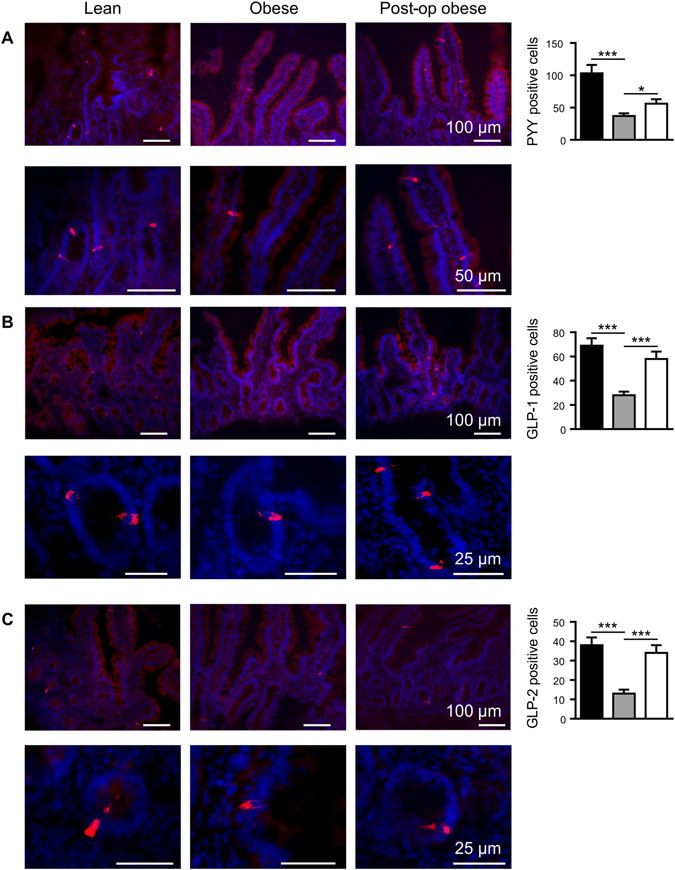
PYY, GLP-1 and GLP-2 protein expression in human duodenal biopsies. Profile of gut hormones, PYY (**A**), GLP-1 (**B**) and GLP-2 (**C**) was determined in the duodenal biopsies of lean (■), obese () and post-operative obese, (□) by immunohistochemistry. Bar charts (on the right) show number of cells counted expressing the gut hormones. Statistical significance was determined by a One-way ANOVA with differences between means identified using a Holm-Sidak multiple comparison post-test test where *p < 0.05, ***p < 0.001. Scale bars are either 100, 50 or 25 µm. Nuclei are stained blue with 4′,6-diamidino-2-phenylindole (Dapi).

By immunohistochemistry we demonstrated that there was a 1.7-fold (p < 0.001) decrease in the total number of EECs in the duodenum of obese patients compared to lean controls (Fig. 1A and Table S1). Using qPCR, a 1.8-fold (p < 0.05) reduction in ChA mRNA expression was also observed in obese duodenal biopsies, compared to lean controls (Fig. 2). The population of EECs in the duodenum, determined by immunohistochemistry, increased 1.6-fold (p < 0.001) 3-month post-operatively, reaching 96% of the total number of EECs observed in lean controls (Fig. 1A). There was 1.8-fold (p < 0.01) decrease in the number of CCK-containing cells in the duodenum of obese compared to lean controls. However, 3 months post-surgery, the number of CCK-expressing EECs in these individuals increased 1.5-fold (p < 0.05) (Fig. 1B). A 2.1-fold (p < 0.001) decline in ghrelin-expressing EECs in the duodenum of obese patients, in comparison to lean, was fully restored post-operatively (p < 0.001) (Fig. 1C). We report here for the first time that human duodenum contains EECs that express PYY, albeit in low numbers. In an average of 100 cells counted, PYY-containing EECs were 2.8-fold (p < 0.001) lower in obese compared to lean controls but displayed a 1.5-fold (p < 0.05) increase post-operatively (Fig. 3A); however the number of PYY-positive cells in the duodenum post-LSG was still 1.8-fold (p < 0.05) lower than lean controls. Furthermore, we show some EECs in human duodenum express GLP-1 and GLP-2, although as expected, the number of GLP-1 and GLP-2 expressing EECs are significantly lower than those expressing CCK or PYY. Duodenal EECs expressing GLP-1 and GLP-2 show a 2.5- (p < 0.001) and 2.9-fold (p < 0.001) reduction, respectively, in obese patients compared to lean controls. Post-operatively the number of GLP-1 and GLP-2 containing EECs increased 2.1- (p < 0.001) and 2.6-fold (p < 0.001), respectively, compared to pre-operative levels (Fig. 3B,C). The findings that that the number of duodenal PYY-containing cells in the post-operative obese group was lower than in lean controls, contrasts with the number of GLP-positive cells in two groups. This may indicate that in human duodenum PYY and GLP-1/2 may be expressed in distinct and separate L-cells. The total number of cells counted that contain ChA and each respective gut hormone are given in the Supplementary Information (Table S1).

### Morphometric analysis of duodenal tissue

By morphometric analysis we observed that there were no differences in villus height/crypt depth in the duodenal tissues of lean, obese and obese individuals after LSG, suggesting that the decline in EEC number is not due to any changes in intestinal surface area (Figure S3). A total of ~250 images each for lean, obese pre-op and obese post-op were used.

### Expression of intestinal epithelial cell markers in the duodenum

**Figure 4:**
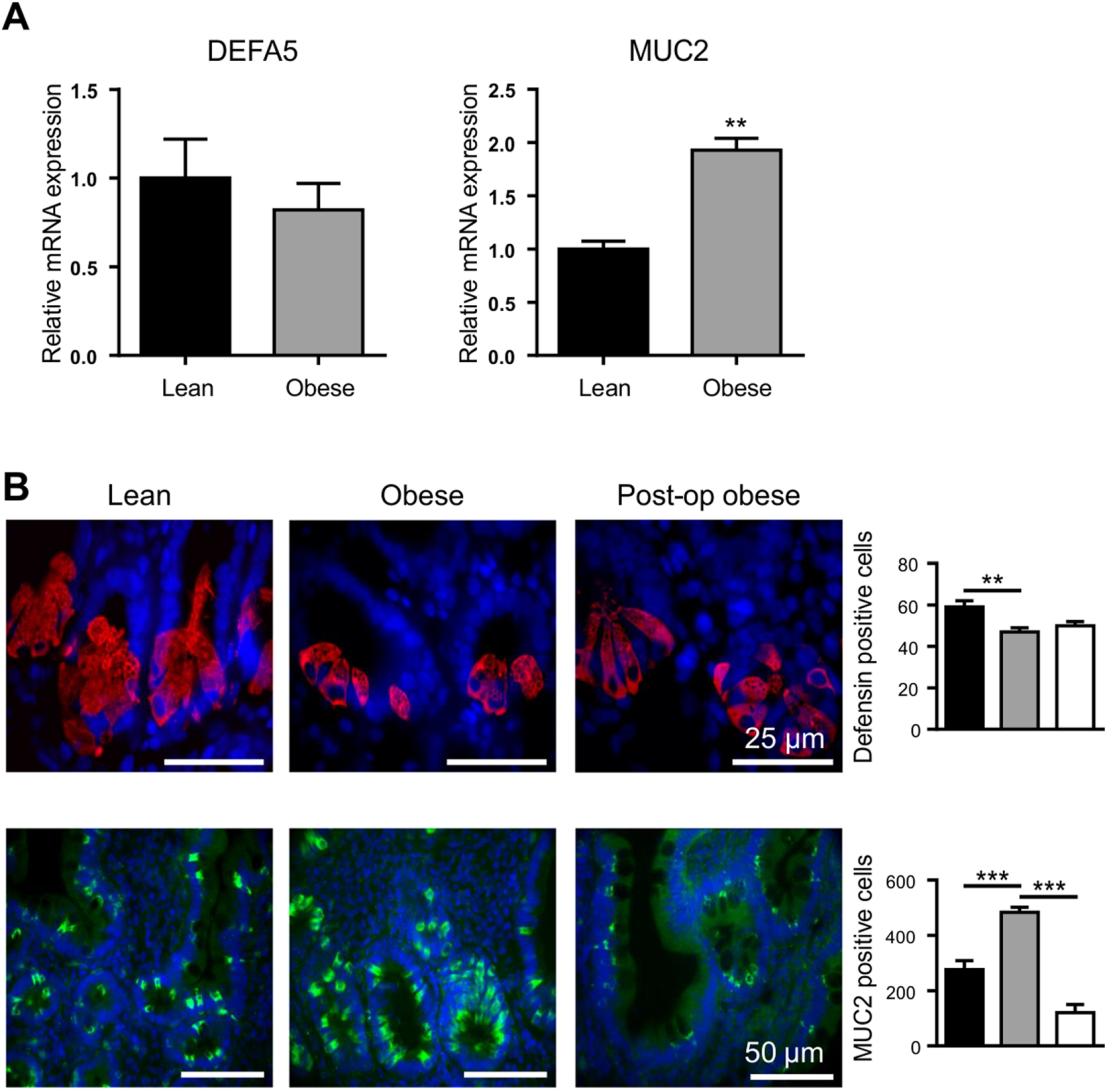
Expression profile of defensin5 and mucin-2 in duodenal biopsies. (**A**) Relative mRNA abundance in lean (■) and obese () duodenum for DEFA5 (Paneth cell marker) and MUC2 (goblet cell marker) determined by qPCR. Values are means ± SEM, normalised to RNA polymerase IIA (POLR2A) m RNA expression, n = 4. (**B**) Profile of defensin 5 (DEFA5; red) and mucin 2 (MUC2; green) determined in lean (■), obese () and post-operative obese, (□) duodenum by immunohistochemistry. Bar charts (on the right) show total number of cells counted expressing either DEFA5 or MUC2. Statistical significance was determined by a Student’s *t*-test (**A**) or One-way ANOVA with differences between means identified using Tukey’s multiple comparison post-test (**B**) where **p < 0.01; ***p < 0.001. Scale bars are 25 µm. Nuclei are stained blue with 4′,6-diamidino-2-phenylindole (Dapi).

**Figure 5:**
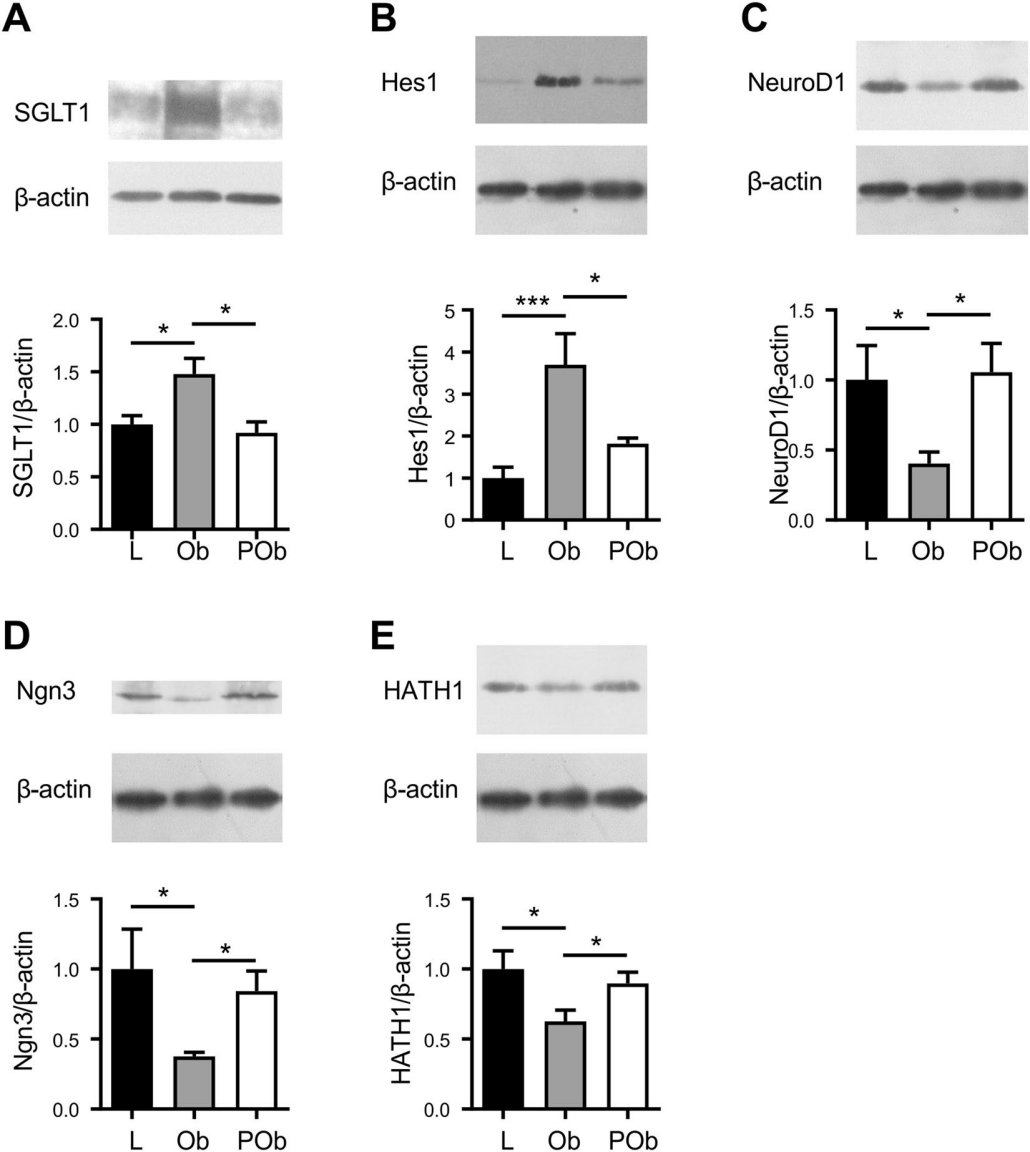
SGLT1, Hes1, NeuroD1, Ngn3, and HATH1 protein expression (western blot) in duodenal biopsies of lean, obese and post-operative. Protein contents of BBMV (20 µg protein), or cell lysates (100 µg protein) separated from duodenal biopsies were subjected to western blot analysis using antibodies to SGLT1 (**A**), transcription factors: Hes1 (**B**), NeuroD1 (**C**), Ngn3 (**D**), HATH1 (**E**) and β-actin; the latter was used as a loading control. Upper panel shows abundance of SGLT1, the transcription factor and β-actin proteins determined by western blots and lower panel depicts the densitometric analysis of western blots normalising protein abundance to that of β–actin: in lean (L) (■), obese (Ob) () and post-operative obese, (POb) (□) individuals. Results are shown as mean ± SEM; n = 7–10. Statistically significant results determined using a One-way ANOVA with differences between means identified using a Holm-Sidak multiple comparison post-test where *P < 0.05 and ***P < 0.001. For clarity, boxed western blot images are displayed. Full length blots are shown in Supplementary Figure S4.

In order to determine whether the alterations in the number of EECs in the intestine of obese subjects may be observed with other intestinal epithelial cells, we determined the expression levels of defensin 5, a marker of Paneth cells^17^ and mucin 2 (MUC2), an indicator of goblet cells^33^ (using qPCR and immunohistochemistry) in duodenal tissues of lean and obese individuals. The protein abundance of Na^+^/glucose co-transporter 1 (SGLT1), a classical marker of absorptive enterocytes, was also assessed (by western blotting). No significant difference in the expression of defensin 5 mRNA was detected in the intestine of obese compared to lean (Fig. 4A), but a moderate 1.3-fold (p < 0.01) decline in cells expressing defensin 5 protein, measured by immunohistochemistry, was observed in obese compared to lean controls (Fig. 4B), and in post-LSG. A marked1.9-fold (p < 0.01) increase in MUC2 mRNA expression (Fig. 4A) and a correlated 1.8-fold increase in MUC2 protein abundance (p < 0.001) (Fig. 4B) were seen in the duodenum of obese compared to lean controls; this increase was reversed 4-fold (p < 0.001), post-operatively (Fig. 4B). The expression of SGLT1 protein was 1.5-fold (p = 0.0114) higher in the intestine of obese compared to lean individuals; and was reversed with a 1.7-fold (p = 0.0262) reduction, post-operatively (Fig. 5A).

### Protein abundance of transcription factors

The abundance of the transcription factors NeuroD1, Ngn3, HATH1 and Hes1 proteins was assessed in duodenal biopsies from lean, obese and obese post-LSG subjects. NeuroD1, Ngn3 and HATH1 protein expression was decreased by 2.5-fold (p = 0.031), 2.7-fold (p = 0.017) and 1.6-fold (p = 0.014) respectively, in obese compared to lean individuals. Conversely, Hes1 protein abundance was 3.4-fold higher (p = 0.001) in the duodenum of obese individuals compared to lean controls (Fig. 5B–E). The observed change in protein abundance of these transcription factors in obese individuals was reversed post-LSG. The levels of NeuroD1, Ngn3 and HATH1 were enhanced 2.9-fold (p = 0.012), 2.3-fold (p = 0.012) and 1.5-fold (p = 0.040), respectively, in post-operative obese duodenum. The increased Hes1 expression seen in obese individuals was reduced by 1.9-fold (p = 0.027) post-LSG, equating to the level detected in the duodenum of healthy controls (Fig. 5B–E).

## Discussion

Gastrointestinal adaptions and alterations in gut hormone secretion levels appear to be key elements in restoring the metabolic improvements seen after bariatric surgery. The two most common and effective procedures are laparoscopic Roux-en-Y gastric bypass (RYGB) and laparoscopic sleeve gastrectomy (LSG). RYGB and LSG produce markedly different anatomical rearrangements. In RYGB a small gastric pouch is formed and the duodenum is bypassed, whilst in LSG a larger portion of the stomach is resected, but the natural passage is unchanged^22^. In spite of these fundamental differences, many clinical and metabolic outcomes (e.g. weight loss, remission of co-morbidities such as type-2 diabetes) are relatively similar after both procedures^26, 34–36^. Different mechanisms have been proposed for explaining the dramatic metabolic improvements. These include calorie restriction, changes in the secretion of incretin, gut hormones and blood glucose levels, accelerated gastric emptying and alterations in bile acid signalling. In RYGB it has been suggested that accelerated travel of nutrients to the distal gut may be a mechanism underlying increased secretion of GLP-1 and PYY^32^. However this mechanism does not entirely explain increased secretion of gut hormones occurring in LSG.

In the present study, we demonstrate a significant increase in EEC numbers after LSG. We show that the decrease in the number of EECs in the obese duodenum is also observed in the stomach. The number of cells expressing ChA, a marker of EECs, was significantly reduced (45%) in the intestine of obese individuals compared to lean controls. This profile was also reflected in the number of cells expressing gut hormones, both in the stomach (ghrelin) and in the duodenum (CCK, GLP-1, ghrelin and PYY). There were no differences in villus height/cryptdepth in the duodenal tissues in obese individuals before and after LSG and to that of control lean subjects, indicating that the decline in the number of EECs in obesity is not due to any structural changes in the intestinal epithelium. It was notable that the population of EECs in obese subjects was increased after LSG, almost reaching the cell numbers seen in the intestine of lean controls.

There are a number of recent publications assessing adaptive response of human or rat intestine after RYGB or LSG. It is important to note that in LSG, one can directly assess structural and functional changes in the same region of the gut (i.e. duodenum) before and after the operation. However, such comparisons cannot be made before and after RYGB, as the duodenum is excluded from nutrient exposure and is difficult to access.

Our study is the first to directly compare structural and functional properties of the same region of human intestine (duodenum) before and after LSG surgery. Our results relate to the finding of Cavin *et al*.^35^ using a “LSG rat model”. They reported that there was no hypertrophy of the jejunal tissue post-surgery, but there was an increased number of GLP-1 containing cells^35^.

Alterations in circulating gut hormone levels in obese versus lean subjects have been published before. It has been reported that postprandial release of GLP-1, PYY and CCK is attenuated, with ghrelin concentrations also being lower in obese compared to lean subjects^10^. After both RYGB and LSG, postprandial release of gut hormones GLP-1, PYY and CCK is increased^10^. The higher number of EECs identified in this study may provide an explanation for this increased gut hormone release.

In LSG, the fundus that contains the majority of ghrelin-releasing cells in the stomach, is resected and therefore, patients show permanently attenuated ghrelin levels without postprandial decline. Slightly higher ghrelin levels observed after 12 months post-operatively compared to 3 months indicate a certain adaption of remaining ghrelin-releasing cells^21^.

Bariatric procedures such as RYGB and LSG are the most effective approaches to resolve type-2 diabetes in obese individuals^36^. This has stimulated research into the role of intestinal glucose absorption in obesity^37, 38^. The intestinal Na^+^/glucose cotransporter 1, SGLT1, is the major route for the absorption of dietary glucose from the lumen of the intestine into absorptive enterocytes^15, 39–43^. Expression and function of SGLT1 is enhanced in the intestine of individuals with type-2 diabetes^44^ and diabetic or obese rodents^45, 46^. SGLT1 overexpression is associated with profound obesity in murine models^47^ proposing an association between increased intestinal glucose transport, diabetes and obesity.

In this study we show that SGLT1 protein abundance is enhanced in the intestine of obese compared to lean controls, and the level is returned to normality after LSG. Nguyen *et al*.^48^, assessing SGLT1 expression at mRNA level, have also reported increased mRNA abundance in the duodenum of obese subjects^48^. It should be borne in mind that assessing expression at both mRNA and protein level will allow determining the cellular level of regulation, i.e. transcriptional, post-transcriptional, or translational. However, it is well established that mRNA expression patterns by themselves are insufficient for the quantitative description of biological systems^49^. Proteins play major roles in the majority of biological processes and measuring protein abundance allows determination of functional relevance.

We observed that protein expression of the transcription factor Hes1, required for absorptive enterocyte differentiation, was increased (compared to lean) in the obese intestine, and normalised post-LSG, mirroring changes in SGLT1 protein abundance.

Our data clearly show that expression levels of the transcription factors HATH1, NeuroD1 and Ngn3, necessary for EEC differentiation, were reduced in obese intestine, in parallel with the number of EECs. However, there is no change in the number of Paneth cells (defensin-5 positive cells) and there is an overexpression of goblet cells (MUC2-labelled cells). Considering that HATH1 specifies the secretory lineage (enteroendocrine, goblet and Paneth cells) our results indicate that there may be alterations in further upstream targets of the HATH1 transcription factor cascade controlling Paneth and goblet cell differentiation in the obese intestine.

Luminal and systemic factors have been implicated in controlling intestinal epithelial cell differentiation. There is compelling evidence that intestinal bacteria can regulate the expression of epithelial cell differentiation factors such as Hes1, Hath1 and KLF4 both *in vivo* and *in vitro*, and that luminal microbiota can directly affect epithelial cell differentiation^50^.

Changes in composition and diversity of gut microbiota have been reported in many pathological conditions leading to altered microbial-host interactions resulting in host malfunctions. For example, there are significant differences in small bowel microbial populations in inflammatory bowel syndrome (IBS) subjects compared to both healthy control and non-IBS individuals^51^. Reduced gut microbial diversity is also a common finding in obesity^52^ leading to modified microbial-host interactions.

With the availability of methodologies to determine changes in mucosa-attached microbiota^53^, one can determine any modifications in duodenal mucosa-attached microbiota before and after surgery. This knowledge will allow a better understanding of factors that may be involved in increased secretion of gut hormones post-surgery allowing the employment of nutritional and/or therapeutic strategies.

In summary, there is a marked reduction in the number of EECs in the duodenum of morbidly obese patients compared to lean controls. LSG surgery results in an increase in the number of EECs to almost normality. We infer from these data that the enhancement in EEC numbers could participate in the markedly increased postprandial gut hormone secretion observed after LSG. Furthermore, the decreased EEC numbers in the intestine of morbidly obese individuals mirrors altered expression of transcription factors controlling epithelial cell differentiation. Deregulation of this regulatory network may lead to defective epithelial differentiation resulting in altered functions of the intestinal epithelium.

Our data support the conclusion that there is a deregulation in intestinal epithelial homeostasis in obesity, which is restored by LSG leading to normalisation of epithelial cell lineage differentiation.

## Experimental Procedures

### Patient selection criteria and the surgical procedure

The protocol was approved by the State Ethics Committee of Basel, Switzerland (EKBB: 298/12) and conducted in accordance with the principles of the Declaration of Helsinki. Patients recruited were those scheduled for bariatric surgery. All participants gave written informed consent. Exclusion criteria for sleeve gastrectomy patients were: presence of any chronic disease of the gastrointestinal tract, and previous gastric surgery. Exclusion criteria for healthy controls were: BMI <18 or >30 kg/m^2^, any chronic illnesses, smoking or substance abuse. Preoperatively study patients followed the standard evaluation for bariatric patients routinely carried out by St. Claraspital and were assessed by an interdisciplinary team consisting of a surgeon, endocrinologist, nutritionist, and a psychiatrist. The preoperative assessment included abdominal ultrasound, upper gastrointestinal x-ray series, gastroscopy and manometry.

The same experienced surgeon successfully completed all procedures laparoscopically. The stomach was dissected by a 6-fold linear stapler beginning 2–4 cm above the pylorus ending at the angle of His over a 35 F bougie. The staple line was over-sewn with an absorbable, monofilament running suture and secured by Lapra-Ty (Ethicon Endo-Surgery, USA) clips.

### Patients, liquid test meal, and plasma removal (Group 1)

Fourteen morbidly obese patients (Table 1) scheduled for LSG were included in this study. There were no perioperative complications. For meal studies, subjects were admitted to the Phase 1 Research Unit of the University Hospital Basel before and 3 months after the operation. After fasting overnight (at least 10 hours), an antecubital vein catheter was inserted and a fasting sample was taken. Then a liquid test meal with a total calorie content of 408 kcal resp. 1708 KJ (Resource diabetes plus 50 g double cream containing 23 g carbohydrates, 15 g proteins and 28 g fat) was served to stimulate hormone release. Blood was drawn at the following times: −15, 0 (corresponding to commencing meal intake), 15, 30, 45, 60, 120 and 180 min. Samples (10 ml/withdrawal) were collected into EDTA tubes containing aprotinin at a final concentration of 500 KIU/ml of blood and a DPP-IV inhibitor; samples were immediately processed and kept on ice to avoid peptide breakdown. After centrifugation at 4 °C, plasma samples were kept frozen at −20 °C until analysis.

### Hormone analysis

The concentrations of cholecystokinin (CCK), glucagon-like-peptide-1 (GLP-1), PYY and insulin were measured in blood plasma using commercially available radioimmunoassay or ELISA kits. The concentration of glucose in the plasma was measured using a commercially available glucoseoxidase method. For further information please see the supplementary methods section.

### Gastrointestinal tissue collection (Group 2)

Thirty-three morbidly obese patients and 24 lean controls were included in the study. Three months post-operatively, 27 out of the 33 obese patients were re-examined (see Table 2) and tissue biopsies were removed as described in supplementary methods (under gastrointestinal tissue collection). Sample processing and analyses were carried out at the University of Liverpool.

### Immunohistochemistry

Tissue biopsies processed into gelatine-embedded blocks were sectioned at a thickness of 10μm, and mounted on polysine-coated slides (Polysine TM, Germany) in preparation for immunohistochemistry. For detection of chromogranin A, mouse monoclonal anti-chromogranin A (K2H10) antibody (1:300, Abcam, Cambridge, UK) or an affinity-purified goat anti-ChrA (E-20): sc-18232 antibody (1:100, Santa Cruz Biotechnology, Santa Cruz, CA) were used. Ghrelin containing cells were identified using an affinity-purified polyclonal rabbit antibody to ghrelin (1:100, bs-0467R, Bioss inc, MA, USA). CCK protein expression was detected using an affinity purified goat polyclonal CCK (C-20) antibody (1:200, sc-21617, Santa Cruz Biotechnology, Santa Cruz, CA). PYY expressing cells were identified using an affinity-purified goat polyclonal peptide YY (N-15) antibody (1:200, sc-47318, Santa Cruz Biotechnology, Santa Cruz, CA). GLP-1 and GLP-2 containing cells were identified by affinity-purified goat polyclonal antibodies, GLP-1 (C-17) and GLP-2 (C-20) (1:100, sc-7782 & sc- 7781, respectively, Santa Cruz Biotechnology, Santa Cruz, CA). Defensin and mucin 2 were selected as markers of Paneth and goblet cells respectively. Cells expressing defensin and mucin 2 were identified by using rabbit monoclonal anti-alpha 5 defensin antibody (EPR14309) (1:3000, Abcam, Cambridge, UK) and an affinity-purified rabbit polyclonal anti-Muc2 antibody (1:40, Sigma-Aldrich Company Ltd., Dorset, UK). Secondary antibodies were used as appropriate and included FITC-conjugated affinity-purified donkey anti-rabbit and anti-mouse IgG and Cy3- conjugated affinity-purified donkey anti-goat IgG (1:500, Jackson ImmunoResearch Laboratories, West Grove, PA). The immunostaining was visualised using an epifluorescence microscope (Nikon, UK) and images were captured with a Hamamatsu digital camera (C4742-95). Specificity of immunostaining was determined by omitting the primary antibody in control sections or by pre-incubating the antibody with an excess of appropriate peptide antigens. The quantification of cell numbers in lesser and greater curvature of stomach as well as in duodenal biopsies of lean, obese- pre-operative or post-operative patients (for ChA, GHR, CCK, PYY, GLP-1, GLP-2 and mucin 2) was performed using a grid (500 × 500 µm^2^), in a defined field of view as described^54^, and cells present on a tissue section of a biopsy within the grid were counted. This approach prevents bias brought by biopsy size. Between 44–1000 immunopositive cells were counted across 5 sections per patient (3–4 biopsies per patient). In the case of defensin, immunopositive cell counting was performed on an average of 10 crypts from 3 different regions of each 3–4 biopsies for each patient.

### RNA isolation and quantitative real-time PCR (qPCR)

mRNA abundance was determined using cDNA prepared from RNA, isolated from duodenal biopsies. Real-time PCR assays were performed using SYBR Green JumpStart Taq ReadyMix for QPCR (Sigma Aldrich). Assays were performed in triplicate using a Rotorgene 3000 (Qiagen, Crawley, UK) and relative abundance was calculated using RG-3000 comparative quantification software. Real-time amplification of RNA polymerase IIA (POLR2A) was carried out simultaneously as the control reference (see Table S2 for qPCR primer sequences).

### Cell lysate isolation

Cell lysates were prepared from two biopsies from the same patient using a buffer consisting of 150 mM NaCl, 10 mM Hepes/Tris pH 7.4, 5 mM EDTA, 1X complete protease cocktail inhibitor [11836-148-001, Roche Diagnostics Ltd, Burgess Hill, UK]). The cellular homogenate was centrifuged at 500 × g (10 min). The final supernatant containing purified cellular lysates was assayed for protein concentration (average lysate volume was 62 ± 11 µl with average protein concentration being 18.6 ± 2.82 µg/µl). The purified cellular lysates were mixed with sample buffer (62.5 mM Tris/HCl pH 6.8, 10% [v/v] glycerol, 2% [w/v] SDS, 0.05% [v/v] β-mercaptoethanol, 0.05% [w/v] bromophenol blue), in preparation for western blotting; with aliquots stored at −20 °C until use.

### Isolation of brush border membrane vesicles

Brush-border membrane vesicles (BBMV) were isolated using Mg^2+^ precipitation/differential centrifugation as previously published^39^. The final pellet containing BBMV were re-suspended in an isotonic buffer (300 mM mannitol, 20 mM Hepes/Tris pH 7.4, 0.1 mM MgSO_4_) at a volume of 10 µl. In preparation for western blot analysis, aliquots of freshly prepared BBMV were assayed for protein content then diluted with sample buffer (see above) and stored at −20 °C until use.

### Western blotting

For the determination of transcription factor protein abundance, protein components of lysates (100 µg) were separated by SDS-polyacrylamide gel electrophoresis on 12% (w/v) polyacrylamide mini gels, containing 0.1% (w/v) SDS, whilst for the determination of SGLT1 protein abundance, protein contents of BBMV (20 µg) were separated using 8% (w/v) polyacrylamide mini gels. Membranes were incubated with either our custom-made antibody to SGLT1^40, 41^ or with antibodies to Neurogenin 3 (ab38548, Abcam, Cambridge, UK), NeuroD1 (ab109224), HATH1 (ab168374) and Hes1 (ab108937). Immunoreactive bands were detected by affinity purified horseradish peroxidase-linked anti-rabbit secondary antibody (DAKO Ltd, Cambridge, UK) and visualised using Immobilon Western Chemiluminescent HRP Substrate (Millipore, Hertfordshire, UK) and Bio-Max Light Chemiluminescence Film (Sigma-Aldrich, Poole, Dorset, UK). The intensity of the immunoreactive bands was quantified using scanning densitometry (Total Lab, Newcastle-upon-Tyne, UK). β-actin protein abundance was used as a loading control.

### Morphometry

Tissue sections were stained with haematoxylin and eosin solutions before dehydration and mounting with D.P.X. neutral mounting medium (Sigma-Aldrich). Digital images were captured with an Eclipse E400 microscope and DXM 1200 digital camera (Nikon, Kingston upon Thames, Surrey, UK), analysed using ImageJ software (Wayne Rasband, US National Institutes of Health, Bethesda, MD) and calibrated using a 100 μm gradient slide. A minimum of three images were captured per section with a minimum of 8 sections prepared per individual, with each section being 5 sections apart within the block. All images were captured under the same conditions. The villus height and the crypt depth measurements were determined blindly taken from an average of sixteen well oriented crypt-villus units per patient.

### Statistics

All parameters were tested for normality by the Shapiro–Wilk test. Descriptive statistics were used for demographic variables such as age, weight, height, and BMI. Hormone profiles were calculated using pharmacodynamic parameters (area under the concentration–time curve (AUC) and maximum plasma concentration (Cmax)). Pre- and post-operative AUC and Cmax of the hormone profiles were compared using Student’s paired *t*-test values. A one-way ANOVA with either a Tukey’s or Holm-Sidak multiple comparison post-test was used to determine the significance of gut hormone, markers of various intestinal epithelial cells and transcription factor abundance. All statistical analyses were carried out using the statistical software package SPSS for Windows Version 19.0 (SPSS Inc., Chicago, USA) or Graphpad Prism for Windows Version 6.0 (Graphpad Inc., CA, USA). Values were reported as mean ± SEM, with p ≤ 0.05 considered as statistically significant.

## Electronic supplementary material

Supplementary material
