# Supplementary material for: Deregulation of transcription factors controlling intestinal epithelial cell differentiation; a predisposing factor for reduced enteroendocrine cell number in morbidly obese individuals
Source: Sci Rep. 2017 Aug 15;7:8174. doi: 10.1038/s41598-017-08487-9 (PMC5557953; doi:10.1038/s41598-017-08487-9)
Supplement: Supplementary file 1 — Supplementary material [file 41598_2017_8487_MOESM1_ESM.doc]

**Deregulation of transcription factors controlling intestinal epithelial cell differentiation; a predisposing factor for reduced enteroendocrine cell number in morbidly obese individuals.**

Bettina K Wölnerhanssen^1,2†^, Andrew W Moran^3†^, Galina Burdyga^3†^, Anne Christin Meyer-Gerspach^1,2†^, Ralph Peterli^4^, Michael Manz^5^, Miriam Thumshirn^5^, Kristian Daly^3^, Christoph Beglinger^1,6^ and Soraya P Shirazi-Beechey^3^*****

*^1^Department of Clinical Research, St. Claraspital Basel, 4058 Basel, Switzerland*

*^2^Department of Biomedicine, University Hospital Basel,* 4056 Basel, *Switzerland*

*^3^Institute of Integrative Biology, University of Liverpool, Liverpool, Merseyside, L69 7ZB, United Kingdom*

*^4^Department of Surgery, St. Claraspital Basel,* *4058 Basel, Switzerland*

*^5^Department of Gastroenterology, St. Claraspital Basel, 4058 Basel, Switzerland*

*^6^Department of Gastroenterology, University Hospital Basel,* 4056 Basel, *Switzerland*

^†^These authors contributed equally to this work

***Corresponding author:** Soraya P Shirazi-Beechey, Institute of Integrative Biology, University of Liverpool, Biosciences Building, Liverpool, L69 7ZB, UK; Email: [spsb@liverpool.ac.uk](mailto:spsb@liverpool.ac.uk)

**Supplementary materials**

1. Gastrointestinal tissue collection

2. Hormone analysis

3. Immunohistochemistry

4. RNA isolation and quantitative real-time PCR (qPCR)

5. Cell lysate isolation

6. Isolation of brush-border membrane vesicles

7. Western blotting

8. Morphometry

9. Supplementary tables and figures

**1. Gastrointestinal tissue collection:**

Thirty-three morbidly obese patients and 24 lean controls were included in the study (Group 2). Three months postoperatively, 27 out of the 33 obese patients were re-examined. Apart from LSG, concomitant cholecystectomy was performed in 4 patients with preoperative cholelithiasis. One patient suffered from upper GI bleeding on postoperative day 1, which could be treated endoscopically, and 1 patient underwent laparoscopic revision for drainage of a hematoma on postoperative day 8. No leakage could be found and the further course was uneventful. There were no other pre-operative complications. Biopsies were removed from three regions of the gastrointestinal tract from lean controls, and morbidly obese individuals pre- and 3 months post-LSG under sedation with intravenous propofol. In addition samples were taken to test for Helicobacter pylori. Three regions were defined: A= lesser curvature, B= greater curvature and C= duodenum pars 2. Four samples were taken at each site from each individual. Postoperatively samples were taken from the duodenum and only the lesser curvature (as the greater curvature had been dissected). After sampling, biopsies were directly immersed in 4% buffered formalin (pH 7.4) and left in this solution at room temperature for 2 hours. Then, the samples were removed and immersed in a 20% sucrose/buffered PBS and stored at 4°C until processing. Further biopsies were placed into cryovials and frozen in liquid nitrogen before storing at -80°C. Fixed and frozen biopsies were sent to Liverpool from Switzerland on ice (4°C) or packed in dry ice respectively. Sample processing and analyses were carried out at the University of Liverpool.

**2. Hormone analysis:**

**Cholecystokinin (CCK)** concentrations were measured by a commercially available radioimmunoassay kit (Euro-Diagnostica AB, Malmo, Sweden). This kit is for assay of CCK in plasma by using an antiserum raised against sulphated CCK-8 N-terminally conjugated to bovine albumin. The antiserum displays 100% cross-reactivity with CCK-8 sulphate, but no relevant cross-reactivity with sulphated gastrin. The intra- and inter-assay coefficient of variation for this assay is below 5.5% and 13.7%, respectively. The lowest detectable concentration is 0.3 pmol/l when using a 200 µl plasma sample. Prior to measurements, CCK was extracted from 1 ml plasma by using an ethanol extraction method. **Glucagon-like-peptide-1 (GLP-1)** was measured employing a commercially available ELISA kit (Millipore Corporation, Billerica, Massachusetts, USA). This kit is for non-radioactive quantification of GLP-1 (7-36) in serum and EDTA-plasma samples; it is highly specific and does not detect other forms of GLP-1. The lowest level of GLP-1 that can be detected by this assay is 0.5 pMol/l when using a 100 μl plasma sample. Prior to measurements, GLP-1 was extracted from 1 ml plasma by using a reversed solid phase extraction (C18-silica cartridges, Waters AG, Baden-Dattwil, Switzerland). **PYY** was measured using a commercially available radioimmunoassay kit (LINCO Research, St. Charles, Missouri, USA). The anti-PYY-antibody used in this kit is raised in guinea pigs and displays 100% cross-reactivity with human PYY1-36 and human PYY3-36, but no cross-reactivity with human pancreatic polypeptide, NPY and unrelated peptides such as leptin and ghrelin. The intra- and inter-assay coefficient of variation for this assay is below 9.4% and 8.5%, respectively. The lowest level of PYY that can be detected by this assay is 10 pg/ml when using a 100 µl plasma sample. **Insulin** was determined using a commercially available radioimmunoassay (CIS bio international, Bagnols, France). This kit is for quantitative determination of insulin in human serum and plasma (EDTA). It is highly specific for insulin and shows no cross-reactivity with other peptides, e.g. c-peptide or glucagon. The intra- and inter-assay coefficient of variation for this assay is below 12.2% and 9.0%, respectively. The lowest level of insulin that can be detected by this assay is 4.6 μU/ml. **Plasma glucose** concentration was measured by a commercially available glucoseoxidase-method (Bayer Consumer Care AG, Basel, Switzerland). This method is highly specific for determination of glucose in serum or plasma. The lowest level of glucose that can be detected by this assay is 0.6 mmol/l.

**3. Immunohistochemistry:**

Frozen gelatine-embedded blocks were sectioned at a thickness of 10 μm, mounted on polysine-coated slides (Polysine TM, Germany) and air-dried. Sections were subjected to heat treatment for 10 min at 60°C, rinsed 3 times in 1 mol/l PBS, permeabilised in a gradient of alcohol and processed for immunohistochemistry. Tissue sections were preblocked by 10% of appropriate blocking reagents and incubated overnight with primary antibodies in PBS with 5% serum. For detection of chromogranin A, mouse monoclonal anti-chromogranin A (K2H10) antibody (1:300, Abcam, Cambridge, UK) or an affinity-purified goat anti-Chr-A (E-20): sc-18232 antibody (1:100, Santa Cruz Biotechnology, Santa Cruz, CA) were used. Ghrelin containing cells were identified using an affinity-purified polyclonal rabbit antibody to ghrelin (1:100, bs-0467R, Bioss Inc, MA, USA). CCK protein expression was detected using an affinity purified goat polyclonal CCK (C-20) antibody (1:200, sc-21617, Santa Cruz Biotechnology, Santa Cruz, CA). PYY expressing cells were identified using an affinity-purified goat polyclonal peptide YY (N-15) antibody (1:200, sc-47318, Santa Cruz Biotechnology, Santa Cruz, CA). GLP1 and GLP2 containing cells were identified by affinity-purified goat polyclonal antibodies, GLP1 (C-17) and GLP-2 (C-20) (1:100, sc-7782 & sc- 7781, respectively, Santa Cruz Biotechnology, Santa Cruz, CA). Defensin and Mucin 2 were selected as markers of Paneth and goblet cells respectively, and were cells containing defensin and mucin 2 were identified using rabbit monoclonal anti-alpha 5 defensin antibody (EPR14309) (1:3000, Abcam, Cambridge, UK) and an affinity-purified rabbit polyclonal anti-Muc2 antibody (1:40, Sigma-Aldrich Company Ltd., Dorset, UK). Secondary antibodies were used as appropriate and included FITC-conjugated affinity-purified donkey anti-rabbit and anti-mouse IgG and Cy3- conjugated affinity-purified donkey anti-goat IgG (1:500, Jackson ImmunoResearch Laboratories, West Grove, PA). Finally, the slides were washed with PBS three times for 5 min and mounted in Vectashield hard set mounting media with DAPI (Vector Laboratories, Burlington, CA, USA). The immunostaining was visualized using an epifluorescence microscope (Nikon, UK) and images were captured with a Hamamatsu digital camera (C4742-95). Specificity of immunostaining was determined by omitting the primary antibody in control sections or by pre-incubating the antibody with an excess of appropriate peptide antigens. The quantification of cell numbers in lesser and greater curvature of stomach as well as in duodenal biopsies of lean, obese- pre-operative or post-operative patients (for ChA, GHR, CCK, PYY, GLP-1, GLP-2 and mucin 2) was performed using a grid (500x500 µm²) (reference 54) and counting cells present on a tissue-section of a biopsy within the grid. Between 44-1000 immuno-positive cells were counted across 5 sections per patient (3-4 biopsies per patient). In the case of defensin, immuno-positive cell counting was performed as an average of 10 crypts from 3 different regions of each 3-4 biopsies for each patient.

**4. RNA isolation and quantitative real-time PCR (qPCR):**

RNA, isolated using the peqGOLD total RNA isolation kit with on-column DNase 1 digestion, (PEQLab, Hampshire, UK), was used as template for first-strand cDNA synthesis. Real-time PCR assays were then performed using 25 ng cDNA as template per 25 µl reaction containing SYBR Green JumpStart Taq ReadyMix for QPCR (Sigma Aldrich) and 900 nM of each primer. PCR cycling was performed as follows: initial denaturation at 95°C for 2 min followed by 30-40 cycles of 95°C for 10 s, 60°C for 30 s. Assays were performed in triplicate using a Rotorgene 3000 (Qiagen, Crawley, UK) and relative abundance was calculated using RG-3000 comparative quantification software. Real-time amplification of RNA polymerase IIA (POLR2A) was carried out simultaneously as a control reference (see below for qPCR primer sequences).

**5. Cell lysate isolation:**

All stages were performed on ice or at 4°C. Two duodenal biopsies from each patient were removed from storage at -80°C and placed onto an ice-cooled glass plate. Using a scalpel, each biopsy was cut up into at least 20 small pieces and transferred to a 1.5 ml microfuge tube on ice. The scalpel was rinsed with 10 µL of lysis buffer (150 mM NaCl, 10 mM Hepes/Tris pH 7.4, 5 mM EDTA, 1X complete protease cocktail inhibitor [11836-148-001, Roche Diagnostics Ltd, Burgess Hill, UK]) and the tissue pieces were homogenised with 20 turns of a loose fitting hand-made glass probe tapered to fit the inside of a 1.5 ml microfuge tube. The probe was rinsed with a 90 µl of lysis buffer into the same 1.5 ml microfuge tube and the suspension further homogenised by passing 40 times through a 19G needle and a further 10 passing through a 23G needle. The homogenate was centrifuged at 10 min at 500 x g (SS34 rotor, Sorvell, UK) and the supernatant transferred to a fresh microfuge tube (average lysate volume was 62 ± 11 µl). The protein concentration in the lysates was estimated by its ability to bind Coomassie blue according to the Bio-Rad assay technique (Bio-Rad, Hemel Hempstead, UK). Porcine γ-globulin was used as the standard (average lysate protein concentration was 18.6 ± 2.82 µg/µl). In preparation for western blot analysis, aliquots of freshly prepared lysates were diluted with sample buffer (62.5 mM Tris/HCl pH 6.8, 10 % [v/v] glycerol, 2 % [w/v] SDS, 0.05 % [v/v] β-mercaptoethanol, 0.05 % [w/v] bromophenol blue) and stored at -20°C until use.

**6. Isolation of brush border membrane vesicles:**

All stages were performed on ice or at 4°C and the methodology was based on the procedure published previously^39^. Two duodenal biopsies from each patient were removed from storage at -80°C and placed onto an ice-cooled glass plate. Using a scalpel, each biopsy was cut up into at least 10 small pieces and transferred to a 1.5 mL microfuge tube on ice. The scalpel was rinsed with 10 µl of a hypotonic buffer (100 mM mannitol, 2 mM Hepes/Tris pH 7.1, 0.5 mM dithiothreitol, 0.2 mM benzamidine and 0.2 mM phenolmethylsulfonyl fluoride)(buffer 1) and the biopsies were homogenised with 10 turns of a loose fitting hand-made glass probe tapered to fit the inside of a 1.5 ml microfuge tube. The probe was rinsed with a 150 µl of buffer 1 into the same 1.5 ml microfuge tube, before attaching the probe to a FUNDAMIX vibro-mixer (DrM, Dr Mueller AG, Maennedorf, Switzerland) and the tissue vibrated (3 x 30 s, setting 7) to remove epithelial cells. MgCl2 was added to the homogenate (10 mM final conc.) and mixed for 20 min at 4°C before centrifuging for 10 min at 3000 x g (SS34 rotor, Sorvell, UK). The supernatant was transferred to a fresh microfuge tube and centrifuged for 45 min at 39000 x g. The final pellet containing BBMV was resuspended in 10 µl of an isotonic buffer (300 mM mannitol, 20 mM Hepes/Tris pH 7.4, 0.1 mM MgSO4) (buffer 2) and the protein concentration was estimated by its ability to bind Coomassie blue according to the Bio-Rad assay technique (Bio-Rad, Hemel Hempstead, UK). Porcine γ-globulin was used as the standard. In preparation for western blot analysis, aliquots of freshly prepared BBMV were diluted with sample buffer and stored at -20°C until use.

**7. Western blotting:**

For the determination of transcription factor protein expression, protein contents of lysates (100 µg) were separated by SDS-polyacrylamide gel electrophoresis on 12% (w/v) polyacrylamide mini gels, containing 0.1% (w/v) SDS, and electrotransferred to polyvinyldifluoride (PVDF) membrane (Immun-Blot, Bio-Rad Laboratories Ltd. Hemel Hempstead, UK). The PVDF membranes were blocked by incubating at RT in TBS-TM (TBS containing 5% (w/v) non-fat dried milk and 0.1% (v/v) Tween-20). Overnight incubation with antibodies to Neurogenin3 (ab38548), NeuroD1 (ab109224), HATH1 (ab168374) and Hes1 (ab108937) diluted 1:1000 in TBS-TM at 4°C then followed. Immuno-reactive bands were detected by incubation for 1 h with affinity purified horseradish peroxidase-linked anti-rabbit secondary antibody (DAKO Ltd, Cambridge, UK) diluted 1:2000 in TBS containing 0.1 (v/v) Tween-20, and visualised using Immobilon Western Chemiluminescent HRP Substrate (Millipore, Hertfordshire, UK) and Bio-Max Light Chemiluminescence Film (Sigma-Aldrich, Poole, Dorset, UK). The intensity of the immunoreactive bands were quantified using scanning densitometry (Total Lab, Newcastle-upon-Tyne, UK). The PVDF membranes blotted with antibodies to HATH1 and Hes1 were stripped by 3 x 10 min washes in 137 mM NaCl, 20 mM glycine/HCl (pH 2.5) and then re-probed with antibodies to NeuroD1 and Ngn3, respectively. Membranes were then stripped again before blotting with monoclonal antibodies to β-actin (clone AC-15, diluted 1:20000; Sigma Aldrich, Poole, Dorset, UK); used as a loading control. Blocking solution consisted of 5 % (w/v) skimmed milk powder, 0.1% (v/v) Triton X-100 and 0.1 mM EDTA in PBS. Incubation and washing buffers were made up of 0.1% (v/v) Triton X-100 and 0.1 mM EDTA in PBS (PBS-TE). Horseradish peroxidase-linked anti-mouse secondary antibody (DAKO Ltd, Cambridge, UK) diluted 1:10000 in PBSTE was used, and visualised as above. For the determination of SGLT1 protein abundance, protein components of BBMV (20 g) were separated on or 8% (w/v) polyacrylamide mini gels and transferred to PVDF membrane, as above. The PVDF membranes were blocked by incubating at room temperature in PBS-TM (PBS containing 0.5% (w/v) non-fat dried milk and 0.1% (v/v) Tween-20) before probing with the custom-made antibody to SGLT1^40,41,44^ in PBS-TM, diluted 1:2000 for 1 hr at RT. Detection of immune-reactive bands was carried out in PBS-TM, as above with β-actin being used as a loading control.

**8. Morphometry:**

Tissue sections were exposed to tap water for 1 min, transferred to Mayer’s Haemalum (3.3 mM Mayer’s Haemalum-haematoxylin, 1 mM sodium iodate, 0.42 mM potassium alum; Sigma-Aldrich, Poole, Dorset, UK) for 1 min and washed gently with running tap water for 5 min. They were stained with eosin Y solution (1 % [w/v] eosin aqueous; HD Supplies, Buckingham, Bucks, UK) for 30 s and subsequently dehydrated by stepwise washing in 70 % ethanol (v/v) for 2 x 1-min, absolute ethanol for 2 x 1-min, and xylene for 3 x 1-min, before mounting with D.P.X. neutral mounting medium (Sigma-Aldrich). Digital images were captured with an Eclipse E400 microscope and DXM 1200 digital camera (Nikon, Kingston upon Thames, Surrey, UK), analysed using ImageJ software (Wayne Rasband, US National Institutes of Health, Bethesda, MD) and calibrated using a 100 μm gradient slide. A minimum of three images were captured per section with a minimum of 8 sections prepared per individual, with each section being 5 sections apart within the block. All images were captured under the same conditions with care taken to ensure that the same villus and crypt were not imaged twice. The crypt depth and the villus height were measured as the average distance from crypt base to crypt-villus junction and villus base to villus tip, respectively. The villus height and the crypt depth measurements were determined blindly from an average of sixteen well oriented crypt-villus units per patient.

**9. Supplementary tables and figures:**

**Table S1:** Protein expression levels of gut hormones and epithelial cell markers in human biopsies before and after LSG.

**Table S2:** qPCR primer sequences.

**Figure S1:** Glucose, insulin and gastrointestinal hormone levels in morbidly obese patients pre- and 3 months post-LSG after liquid test meal (408 kcal) stimulation (Group 1).

**Figure S2:** Chromogranin A and ghrelin in human greater and lesser curvature of stomach.

**Figure S3:** Morphometric analysis of crypt-depth, villus height in duodenal biopsies of lean, obese and post-operative obese subjects.

**Figure S4:** Full length images of western blots shown as boxed in Figure 5.

**Table S1: Protein expression levels of gut hormones and epithelial cell markers in human biopsies before and after LSG.**

| **Hormone** | **Site** | **Number of cells counted** | | |
| --- | --- | --- | --- | --- |
|  |  | **Lean** | **Obese pre-op** | **Obese post-op** |
| **ChA** | A  B  C | 909 ± 69  910 ± 70  692 ± 47 | 405 ± 30***  394 ± 25***  413 ± 22*** | 640 ± 74*  -  665 ± 60*** |
| **GHR** | A  B  C | 504 ± 42  573 ± 60  250 ± 18 | 204 ± 18***  206 ± 20***  121 ± 3*** | 331 ± 61*  -  241 ± 17*** |
| **CCK** | A  B  C | 0  0  264 ± 27 | 0  0  145 ± 6** | 0  -  211 ± 13* |
| **PYY** | C | 103 ± 13 | 37 ± 4*** | 56±7* |
| **GLP-1** | A  B  C | 0  0  69 ± 6 | 0  0  28 ± 3*** | 0  -  58 ± 6*** |
| **GLP-2** | A  B  C | 0  0  38 ± 4 | 0  0  13 ± 2*** | 0  -  34 ± 4*** |
| **Defensin** | C | 59 ± 3 | 47 ± 2** | 50 ± 2 |
| **Muc-2** | C | 276 ± 33 | 484 ± 18*** | 121 ± 29*** |

A - lesser curvature; B - greater curvature; C – duodenum. ChA - chromogranin A; GHR – ghrelin; CCK – cholecystokinin; PYY – peptide YY; GLP1- glucagon like peptide 1; GLP2 - glucagon like peptide 2; Muc-2 - mucin 2. ***p < 0.001; **p < 0.01; *p < 0.05

**Table S2: qPCR primer sequences**

| **Name** | **Sequence** |
| --- | --- |
| Human ChA S | CAGCCCCACAACTTTAAACATTG |
| Human ChA AS | GGATGGAGGAAGGGAAACTTCTAG |
| Human DEFA5 S | GCAGTCTGGGGAAGACAACC |
| Human DEFA5 AS | CAGGACCTTGAACTGAATCTTGC |
| Human MUC2 S | GATCCAGCGTGATGAGGGTC |
| Human MUC2 AS | TGGAGCGGTGGTCAAAGTTC |
| Human POLR2A S | GCAAGCGGATTCCATTTGG |
| Human POLR2A AS | TCTCAGGCCCGTAGTCATCCT |

**
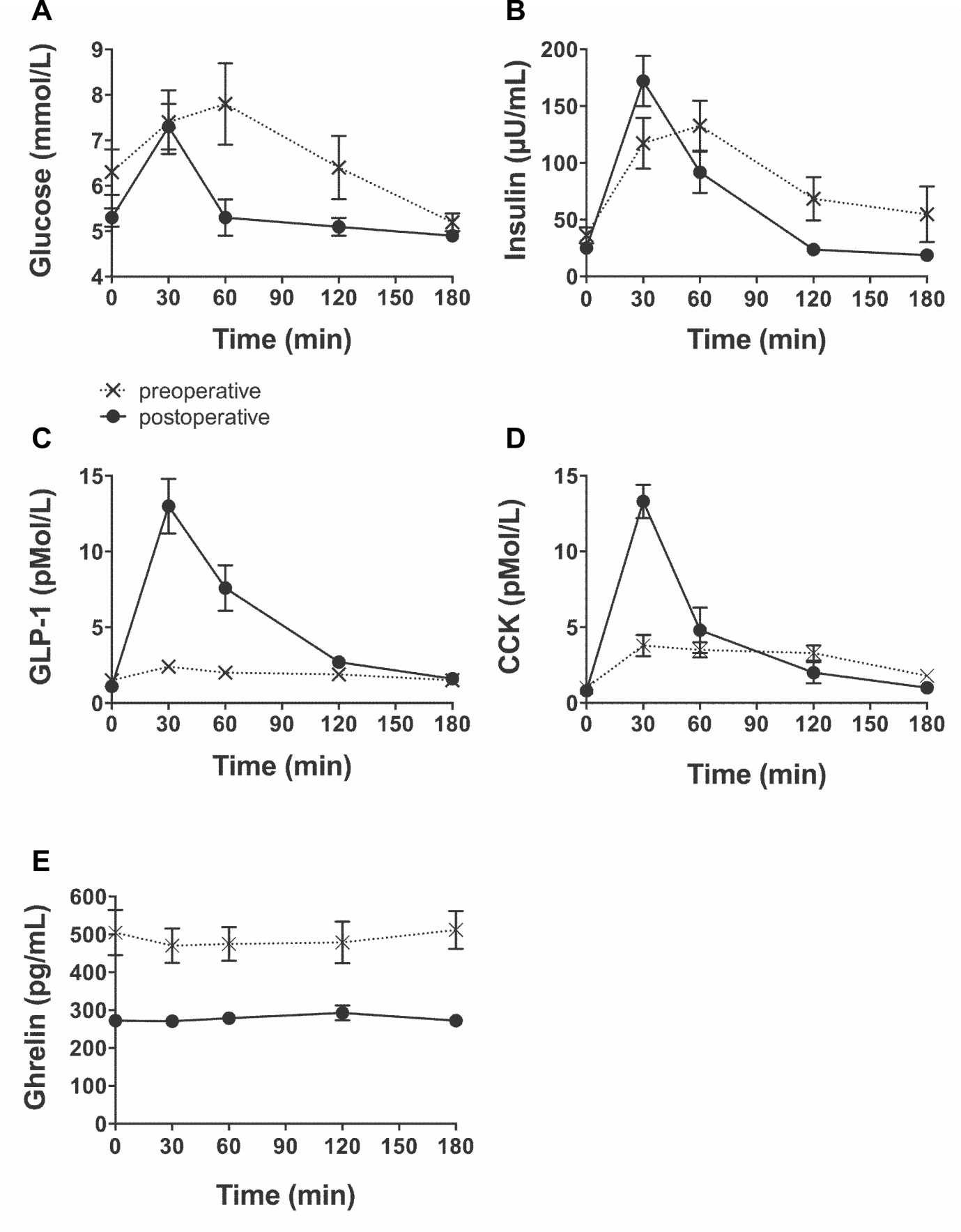
Figure S1: Glucose, insulin and gastrointestinal hormone levels in morbidly obese patients pre- and 3 months post-LSG after liquid test meal (408 kcal) stimulation (Group 1).** The concentration of glucose (**A**), insulin (**B**) and the gastrointestinal hormones GLP-1 (**C**), CCK (**D**) and ghrelin (**E**) were determined in the blood before and at intervals after pre- (dotted lines) and post-operative (solid line) patients were administered a liquid test meal. Data represents mean ± SEM.

**
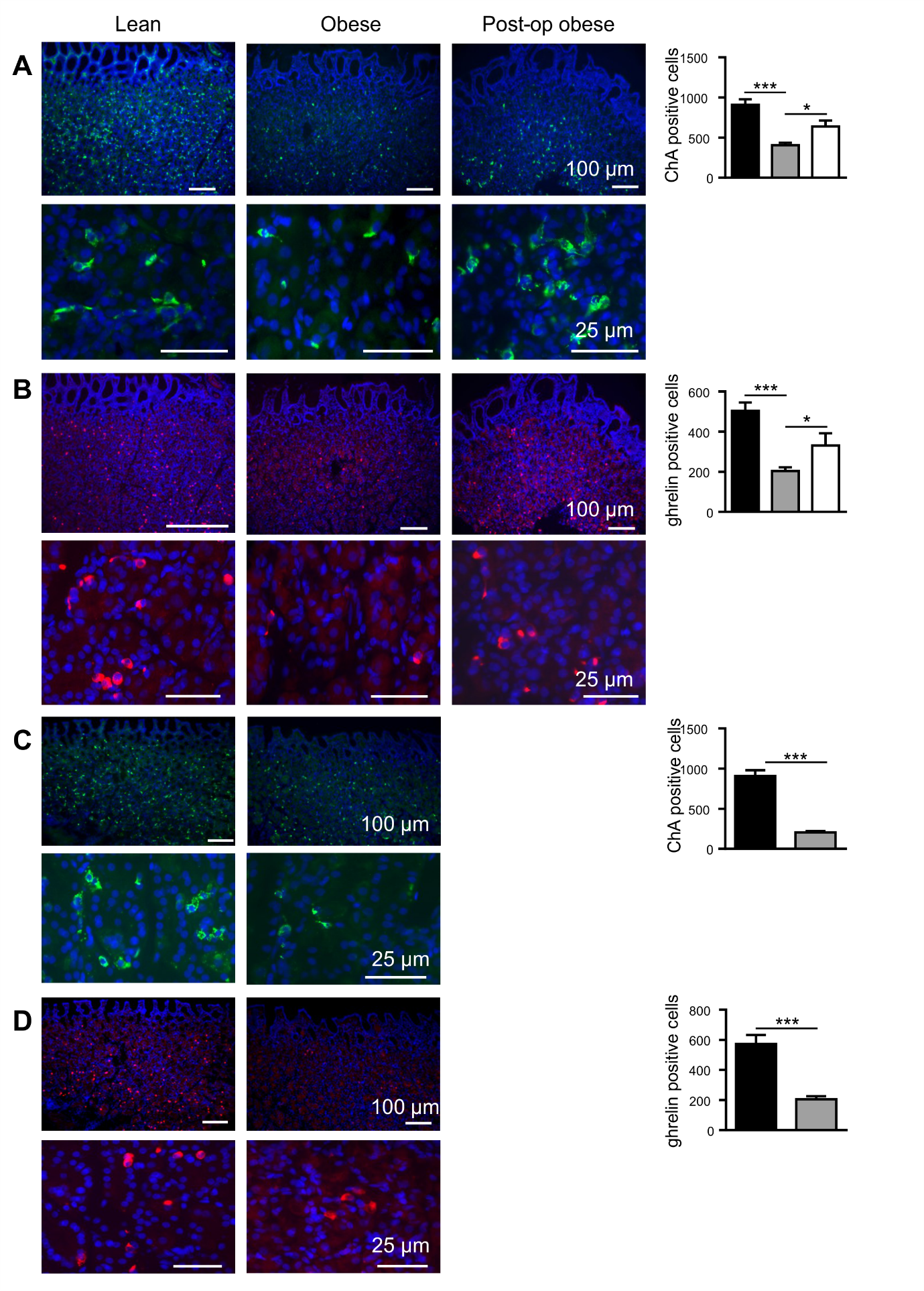
Figure S2: Chromogranin A and ghrelin in human greater and lesser curvature of stomach.** Protein expression of the enteroendocrine cell marker, chromogranin A (**A** & **C**, ChA) and ghrelin (**B** & **D**) was determined in the lesser (**A** & **B**) and greater (**C** & **D**) curvature of the stomach in lean, obese and post-operative obese (lesser curvature) patients by immunohistochemistry. Bar charts (right) show number of cells counted expressing either ChA or ghrelin. Statistical significance was determined by a One-way ANOVA with differences between means identified using a Holm-Sidak multiple comparison post-test (A & B) or a Student’s *t*-test (C & D) where **p < 0.01 and ***p < 0.001. Scale bars are 25 µm. Nuclei are stained blue with 4',6-diamidino-2-phenylindole (Dapi).

**
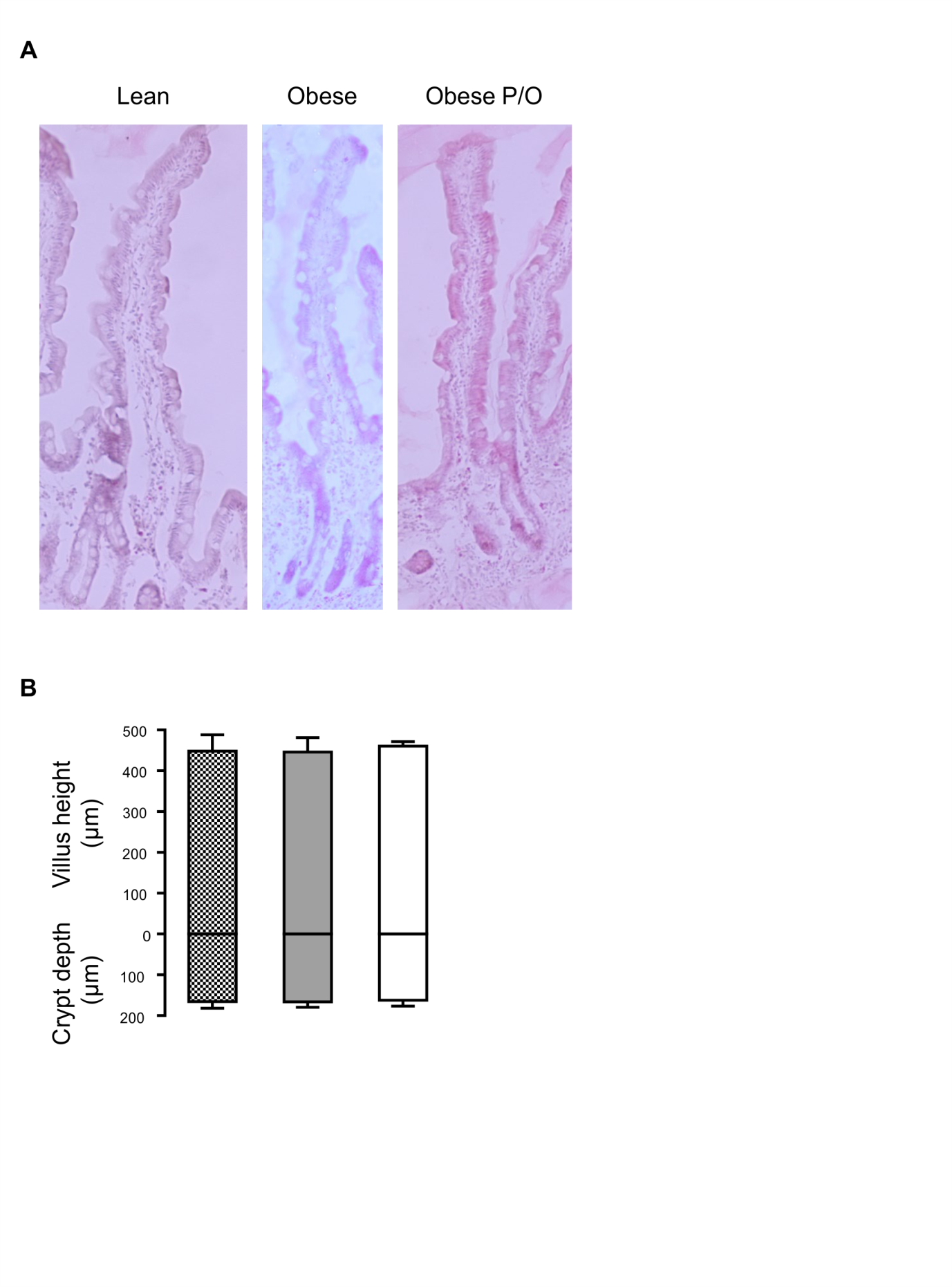
Figure S3: Morphometric analysis of crypt-depth, villus height in duodenal biopsies of lean, obese and post-operative obese subjects.** (**A**) Representative light micrographs showing crypt depth and villus height measured in duodenal tissues of lean, obese and post-operative obese patients. Images were obtained at 10X magnification. (**B**) Morphometric analyses of villus height and crypt depths are shown as histograms, in (µm) ± SD. Lean (), obese () and postoperative obese ( post-op obese); n=5-12. Statistically significance was determined using a one-way ANOVA.


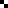


**
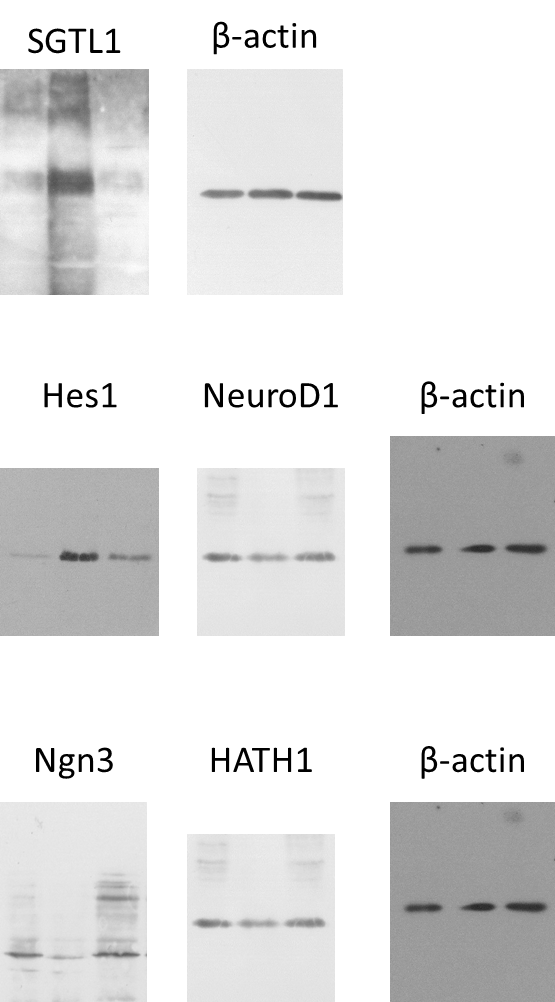
**

**Figure S4: Full length images of western blots shown as boxed in Figure 5.**
